# The effect of unemployment on suicidal ideation among men: evidence from Australia during the COVID-19 pandemic

**DOI:** 10.3389/fpubh.2025.1544151

**Published:** 2026-02-19

**Authors:** Andrew R. Timming

**Affiliations:** College of Business, Alfaisal University, Riyadh, Saudi Arabia

**Keywords:** COVID-19, health, job loss, mental illness, pandemic, suicidal ideation

## Abstract

**Introduction:**

The COVID-19 pandemic presented governments with unprecedented choices that had to be made under conditions of imperfect information. One of the key trade-offs pertains to decision-making around social distancing mandates to reduce the risk of infection, resulting in high levels of joblessness. Against this backdrop, the present study examines the relationship between COVID-19-related job loss and suicidal ideation among Australian men.

**Methods:**

Using the *Ten to Men* dataset, we use multivariate logistic regression analysis to unpack the change in odds of suicidal ideation across eight additive models. This dataset used a stratified, multi-stage cluster sampling technique, and the final sample size is *n* = 963. A large number of control variables were introduced sequentially to address endogeneity and prevent omitted variable bias.

**Results:**

COVID-19-related job loss in Australia is significantly associated with suicidal ideation in our full model. Men who were made unemployed during the pandemic were 2.77 times more likely to have suicidal thoughts than men who remained in employment.

**Conclusion:**

These odds are much higher than meta-analytic evidence of the relationship between pre-COVID-19 unemployment and suicidal ideation, suggesting that unemployed Australian men experienced high levels of suicidal thoughts during the pandemic. The Australian Government's wage subsidy program, JobKeeper, may have provided a financial social safety net, but our research suggests, uniquely, that it was the loss of work relationships, not the loss of income, that is the main concern. Work, for men, is not just a paycheck; it is a source of meaning and provides a purpose in life.

## Introduction

The global transmission of the SARS-CoV-2 virus in 2020 presented governments and policy-makers with difficult decisions that were made under circumstances of imperfect information. Based on an over-estimation of the infection mortality rate (IFR) (1), the public health response of many governments (2) was to impose mandatory stay-at-home lockdowns to promote social distancing and obviate transmission, a strategy whose effectiveness is still being assessed today (3). It soon became evident that the lockdown restrictions entailed various “collateral damages” (4), including potentially adverse impacts on mental health and wellbeing (5). Men appear to have suffered from especially high levels of “economic anxiety”—driven by fear of job loss—in the face of these restrictions (6).

Within this body of literature on the relationship between the COVID-19 pandemic and mental health/illness, limited scholarly attention has been paid to suicide and suicidal ideation (7–11) and associated preventive measures (12, 13). Most studies explain suicidal thoughts and behaviors as a function of COVID-19 restrictions' impact on mental health, loneliness, or domestic abuse (14). Only a handful of studies have zeroed in on the effect of COVID-19-related job loss on suicidal ideation [e.g., (15)]. Unemployment among men is particularly relevant since joblessness is likely to challenge their sense of masculinity (16). Meta-analytic evidence suggests that unemployment significantly increases the odds of suicide mortality, attempts, and ideation (17). The prevailing theory is that the state of being unemployed is associated with high levels of psychosocial distress, which is driven by perceived low social status (18) as well as a strong sense of financial uncertainty and vulnerability (6). In short, the unique contribution of the present study to the extant literature is that, unlike previous studies in this area (15), we focus exclusively on men's mental health, with particular attention paid to the role of job loss in promoting suicidal ideation during the pandemic. To achieve this contribution, empirical analysis is required.

To this end, using the latest data release from the Ten to Men project (19), the present study seeks to examine the relationship between COVID-19-related job loss and suicidal ideation, while controlling for several correlates. It is hypothesized that unemployment stemming directly from lockdown restrictions will be strongly associated with suicidal thoughts. Drawing from an Australian dataset adds further value to these analyses because Australia experienced one of the longest lockdowns in the world (20). Citizens were mandated to stay at home for months. To mitigate against the economic harm associated with this public health policy, the government implemented a “JobKeeper” subsidy to “freeze” the economy so that it could then be swiftly un-thawed at the conclusion of the pandemic (21). This program, which provided wage subsidies to businesses and millions of employees, cost approximately $89B, amounting to 4.5% of Australia's GDP (22). This stimulus package may have further mitigated against suicidal ideation, though limited research corroborates this claim.

To address endogeneity and prevent omitted variable bias, relevant control variables were introduced into the analyses. These controls are commonly thought to be associated with suicidal ideation and sub-optimal mental health. The controls included in the present study are: job satisfaction (23), aboriginality (24), living alone (25), age (26), mental health/illness and subjective wellbeing (27), physical health (28), alcohol consumption (29), optimism/pessimism (30), and perceived unfair treatment based on individual/group differences [e.g., (31–34)]. The inclusion of these covariates enables us to isolate the association between COVID-19-related job loss and suicidal ideation.

This research is important because of the catastrophic consequences that suicide has on society (35). In Australia alone, 2,358 men died by their own hand in 2021 (36), amounting to tens of thousands of deaths across the last several decades. More importantly, the value-added of the present study is its unique focus on Australian men and their state of mind in the midst of the COVID-19 epidemic. Moreover, the backdrop of Australia's wage subsidy program also offers fertile ground for examining the economics of suicide in the context of a high-stress event such as the pandemic. If the Government's lockdown response contributed in any way to suicidal thoughts and behaviors, then lessons must be learned to prepare ourselves for a future global health crisis.

## Data and methods

### Sampling

The study's hypothesis was tested using the latest Wave 3 data from “Ten to Men: The Australian Longitudinal Study on Male Health,” a dataset compiled across 2020–2021 by the Australian Institute of Family Studies (19, 37). The Ten to Men dataset examines six research “domains” pertaining to male health, including: (1) wellbeing and mental health, (2) use of health services, (3) health-related behaviors, (4) health status, (5) health knowledge, and (6) social determinants of health. In light of the pandemic, the Wave 3 data were collected via contactless (i.e., online) interviewing (ibid.: 3).

The Ten to Men dataset was compiled using a stratified, multi-stage cluster probability sample [for more information on the methodology, see Ref. (38)]. The Wave 3 sample used in this study consists of *n* = 7,919 respondents and has an overall response rate of 53% (39). Missing data were coded from −8 to −1 and excluded from all analyses, along with listwise deletion of cases and the exclusion of children. Although listwise deletion can result in bias insofar as the data are not missing completely at random (40), any such bias is unlikely given the careful, systematic approach to data collection in Wave 3. Response bias, however, is an ever-present threat for self-reported data such as these. The final sample size for the present study is *n* = 963, which is due to the removal of missing data, children, and individuals who were not asked about suicidal thoughts and behaviors.

To bolster validity and replicability, several established inventories were used to measure the variables, including, for example, the widely used Alcohol Use Disorders Identification Test (AUDIT) and the Personal Wellbeing Index (PWI), both of which have been found to have high levels of reliability (i.e., Cronbach's alpha >0.800) as well as good construct validity [see Ref. (41, 42) respectively]. For purposes of replication, a full list of the inventories can be found in the Australian Institute of Family Studies (43).

Sample demographics are reported in Appendix A.

### Study variables

Table 1 in Appendix B reports the variables used in the present study.

*Criterion variable*. The criterion variable, suicide, was measured by asking the respondents if they had any suicidal thoughts over the past 12 months (0 = no, 1 = yes).

*Primary predictor variable*. The primary predictor variable, COVID-19 job loss, was measured by asking the respondents if they had lost their job at any time over the last 12 months (0 = no, 1 = yes).

*Covariates*. Several covariates were included sequentially and additively across the models, as articulated in Table 1 in Appendix B.

### Statistical technique

Stepwise multivariate logistic regression analysis was used to predict suicidal ideation, with all variables retained at each step. The final model was checked for multicollinearity, and no VIF statistics exceeded the common threshold of 10 [(44); p. 272], suggesting no problems with this assumption. For a full description of the method (see Appendix C).

## Results

Step 1 examines the bivariate relationship between COVID-19 job loss and suicide. The results suggest that COVID-19 job loss is positively associated with suicidal thoughts (*B* = 0.62, S.E. = 0.24, Wald = 6.53, *p* = 0.01). Respondents who experienced job loss during the pandemic are 1.85 times (95% C.I. = 1.15 to 2.97 times) more likely to have had suicidal thoughts in the past 12 months than respondents who did not experience job loss during the pandemic.

Step 2 examines the relationship between COVID-19 job loss and suicide, controlling for job satisfaction. COVID-19 job loss is still positively associated with suicidal thoughts (*B* = 0.78, S.E. = 0.34, Wald = 5.37, *p* = 0.02). Respondents who experienced job loss during the pandemic are now 2.18 times (95% C.I. = 1.23–4.20 times) more likely to have had suicidal thoughts in the past 12 months than respondents who did not experience job loss during the pandemic. Job satisfaction also significantly predicts suicidal thoughts (*B* = −0.22, S.E. = 0.04, Wald = 35.07, *p* < 0.01, Exp(*B*) = 0.80, 95% C.I. = 0.74–0.86).

Step 3 examines the relationship between COVID-19 job loss and suicide, controlling additionally for the following individual differences: whether the respondent is aboriginal, whether the respondent is living alone, and the respondent's age. COVID-19 job loss is still positively associated with suicidal thoughts (*B* = 0.73, S.E. = 0.04, Wald = 4.54, *p* = 0.03) in the past 12 months. Respondents who experienced job loss during the pandemic are now 2.07 times (95% C.I. = 1.06–4.03 times) more likely to have had suicidal thoughts than respondents who did not experience job loss during the pandemic. Job satisfaction still significantly predicts suicidal thoughts (*B* = −0.22, S.E. = 0.04, Wald = 33.48, *p* < 0.01, Exp(*B*) = 0.80, 95% C.I. = 0.74–0.86). Living alone furthermore predicts suicidal thoughts (*B* = 0.72, S.E. = 0.22, Wald = 11.19, *p* < 0.01, Exp(*B*) = 2.06, 95% C.I. = 1.35–3.15), as does age (*B* = −0.02, S.E. = 0.01, Wald = 7.23, *p* = 0.01, Exp(*B*) = 0.98, 95%C.I. = 0.97–1.00).

Step 4 examines the relationship between COVID-19 job loss and suicide, controlling additionally for the following mental health variables: depression in the past 12 months, anxiety in the past 12 months, other mental health conditions in the last 12 months, risk-taking score, and subjective wellbeing score. In this model, COVID-19 job loss is not significantly (though it is marginally) related to suicidal thoughts in the past 12 months (*p* = 0.06). Three of the covariates significantly predict the outcome: age (*B* = −0.02, S.E. = 0.01, Wald = 6.40, *p* = 0.01, Exp(*B*) = 0.98, 95%C.I. = 0.97–1.00), depression in the past 12 months (*B* = 0.85, S.E. = 0.19, Wald = 19.68, *p* < 0.01, Exp(*B*) = 2.35, 95%C.I. = 1.61–3.42), and subjective wellbeing score (*B* = −0.03, S.E. = 0.01, Wald = 46.24, *p* < 0.01, Exp(*B*) = 0.97, 95%C.I. = 0.96–0.98).

Step 5 examines the relationship between COVID-19 job loss and suicide, controlling additionally for the following set of physical health indicators: self-rated health, minutes of sleep each weekday night, minutes of sleep each weekend night, BMI, and whether the respondent has been diagnosed with a health condition in the past 12 months. COVID-19 job loss is once again positively associated with suicidal thoughts in the past 12 months (*B* = 0.78, S.E. = 0.37, Wald = 4.58, *p* = 0.03). Respondents who experienced job loss during the pandemic are now 2.18 times (95% C.I. = 1.07–4.56 times) more likely to have had suicidal thoughts than respondents who did not experience job loss during the pandemic. Two of the remaining covariates predict suicidal thoughts: depression in the past 12 months (*B* = 0.89, S.E. = 0.23, Wald = 14.91, *p* < 0.01, Exp(*B*) = 2.53, 95% C.I. = 1.55–3.80) and subjective wellbeing score (*B* = −0.03, S.E. = 0.01, Wald = 32.25, *p* < 0.01, Exp(*B*) = 0.97, 95% C.I. = 0.96–0.98).

Step 6 examines the relationship between COVID-19 job loss and suicide, controlling additionally for alcohol consumption score. COVID-19 job loss is again positively associated with suicidal thoughts in the past 12 months (*B* = 0.78, S.E. = 0.37, Wald = 4.55, *p* = 0.03). Respondents who experienced job loss during the pandemic are 2.18 times (95% C.I. = 1.07–4.45 times) more likely to have had suicidal thoughts than respondents who did not experience job loss during the pandemic. The same two covariates from Step 5 again predict suicidal thoughts: depression in the past 12 months (*B* = 0.88, S.E. = 0.23, Wald = 14.80, *p* < 0.01, Exp(*B*) = 2.42, 95% C.I. = 1.54–3.79) and subjective wellbeing score (*B* = −0.03, S.E. = 0.01, Wald = 31.96, *p* < 0.01, Exp(*B*) = 0.97, 95% C.I. = 0.96–0.98).

Step 7 examines the relationship between COVID-19 job loss and suicide, controlling additionally for eight measures of optimism toward life overall, employment stability, financial situation, housing, relationships, mental health, physical health, and ability to adapt to challenges. In this model, COVID-19 job loss is not significantly (though it is marginally) associated with suicidal thoughts in the past 12 months (*p* = 0.08). The following covariates significantly predict suicidal thoughts: depression in the past 12 months (*B* = 0.73, S.E. = 0.25, Wald = 8.92, *p* < 0.01, Exp(*B*) = 2.08, 95% C.I. = 1.29–3.35), subjective wellbeing score (*B* = −0.02, S.E. = 0.01, Wald = 5.73, *p* = 0.02, Exp(*B*) = 0.98, 95% C.I. = 0.97–1.00), optimism about relationships (*B* = −0.25, S.E. = 0.09, Wald = 7.03, *p* = 0.01, Exp(*B*) = 0.78, 95% C.I. = 0.65–0.94), optimism about mental health (*B* = −0.61, S.E. = 0.12, Wald = 25.14, *p* < 0.01, Exp(*B*) = 0.54, 95%C.I. = 0.43–0.69), and optimism about physical health (*B* = 0.25, S.E. = 0.12, Wald = 4.56, *p* = 0.03, Exp(*B*) = 1.28, 95% C.I. = 1.02–1.60).

Finally, Step 8 examines the relationship between COVID-19 job loss and suicide, controlling additionally for 11 measures of perceived unfair treatment based on language or accent, skin color, age, disability, religious beliefs, cultural background, mental health problems, sexual identity, body appearance, sex, and gender. COVID-19 job loss is once again positively associated with suicidal thoughts in the past 12 months (*B* = 1.02, S.E. = 0.45, Wald = 5.07, *p* = 0.02). Respondents who experienced job loss during the pandemic are now 2.77 times (95% C.I. = 1.14–6.70 times) more likely to have had suicidal thoughts than respondents who did not experience job loss during the pandemic. Four of the remaining covariates significantly predict suicidal thoughts: depression in the past 12 months (*B* = 0.68, S.E. = 0.26, Wald = 7.06, *p* = 0.01, Exp(*B*) = 1.97, 95% C.I. = 1.19–3.24), subjective wellbeing score (*B* = −0.02, S.E. = 0.01, Wald = 6.40, *p* = 0.01, Exp(*B*) = 0.98, 95% C.I. = 0.96–1.00), optimism about relationships (*B* = −0.25, S.E. = 0.10, Wald = 6.22, *p* = 0.01, Exp(*B*) = 0.78, 95% C.I. = 0.65–0.95), and optimism about mental health (*B* = −0.61, S.E. = 0.13, Wald = 22.49, *p* < 0.01, Exp(*B*) = 0.55, 95% C.I. = 0.43–0.70).

The best way to interpret these results is that, though the inputs toward suicidal ideation during the pandemic are complex and multifaceted, the evidence overwhelmingly suggests that job loss, in and of itself, strongly predicts suicidal thoughts among Australian men. Table 2 in Appendix D reports the results of the full multivariate model at Step 8 with all controls.

## Discussion and conclusion

The relationship between COVID-19-related job loss and suicidal ideation remains significant even after the inclusion of all of the controls. According to Amiri's (17) meta-analysis of 54 peer-reviewed studies, the likelihood of suicidal thoughts among the unemployed is 85% higher than found in the general employed population. In the present study, the increase of suicidal ideation among unemployed Australian men is 177% higher (Exponentiated(*B*) = 2.77). One possible reason for the comparatively higher levels of suicidal ideation is that pre-COVID-19 job loss may be substantively different in scope and impact vis-à-vis COVID-19-related job loss. Men who found themselves unemployed as a result of COVID-19 faced not only job loss, but the “double whammy” of a pandemic. This combination of job loss, alongside health fears, may have exacerbated suicidal ideation. Thus, in terms of the mechanism at play, epidemiological factors may interact with loss of work-related relationships, such that they contribute to psychosocial distress more powerfully together than they do apart. This finding is consistent with recent research demonstrating the additive effect of multiple stressors (45).

What makes our results particularly interesting is that they imply—at least implicitly—that the effect of unemployment on suicidality may have less to do with financial vulnerability and uncertainty than was previously thought. As noted above, the pandemic in Australia was met with a historically unprecedented stimulus package commonly referred to as “JobKeeper” (21). So significant were these wage subsidies that it has even been estimated that poverty and housing stress were *lower* under COVID-19 than they were before the pandemic (46). In short, COVID-19-related job loss did not result in increased economic precarity, a key input to previous modeling of suicidal ideation (47); our evidence points to the opposite effect.

One explanation for this ostensibly paradoxical effect is that the loss of work under the pandemic had less to do with financial loss and more to do with the loneliness (48) associated with the loss of work relationships and the depletion of “belongingness” (49). To be sure, this explanation is consistent with, and aligned to, the fact that “relationships optimism,” as a covariate, was significantly negatively related to suicidal ideation in the full model. It is well-known that labor is about much more than just a paycheck, and that individuals derive dignity and deep meaning from their work. Further empirical and theoretical research is needed to confirm and unpack these effects.

The results of our research have important implications for public policy. As the pandemic recedes into history, now is an ideal time to take stock of the purported efficacy, or lack thereof, of the lockdown measures so that we can evaluate whether the “cure” was worse than the “disease” (50, 51). The question of whether the trade-off between jobs and public health is utilitarian is an empirical one, with evidence both in favor of lockdowns (52) and against (53). What is needed—certainly in Australia and perhaps elsewhere—is a non-partisan public inquiry into the lockdown response, wherein transdisciplinary experts come together to present evidence.

In spite of the robust study design, extensive use of control variables, and high-quality data, the research presented here has a number of limitations, each of which lends itself to a direction for future research. First, these data are cross-sectional, meaning that, at best, we can speak of associations among our variables. To establish causality, data collection at multiple points in time is needed. Suicidal ideation, by nature, is dynamic and cannot easily be understood cross-sectionally. Second, the study focuses on suicidal thoughts, not behaviors. Future research might do well to predict suicidal planning and attempts. Third, the results are confined to a single country—Australia—and a single gender—men. Further cross-national research is urgently needed to establish the generalizability of the results and the “overall disease burden” (54) across the world. Fourth, sample selection bias, particularly in relation to self-selection, and response bias, especially in respect to social desirability, are ever-present threats that must always be borne in mind in the interpretation of the results of this research. It is difficult to minimize these biases given the controversial nature of research on suicide. Fifth, and preceding from the aforementioned limitation, it needs to be recognized that measurement error and recall bias are potential pitfalls to the extrapolation of the present study's results to the wider population of Australian men. Sixth, the stepwise nature of the modeling could potentially make the relationships more susceptible to suppression effects among predictors. In this case, it is possible that significant relationships are due to the presence of the effect of covariates. This is an inherent limitation in any multivariate model and can be remedied by further research examining different configurations of predictors. Finally, with regard to the 99.4% male statistic, this can be explained by the increasing gender fluidity over the last few years. A small minority of men now identify as female or non-binary, but, unfortunately, the survey did not provide respondents with those options.

## Data Availability

The original contributions presented in the study are included in the article/supplementary material, further inquiries can be directed to the corresponding author.
