# Supplementary material for: The effect of unemployment on suicidal ideation among men: evidence from Australia during the COVID-19 pandemic
Source: Front Public Health. 2026 Feb 19;13:1544151. doi: 10.3389/fpubh.2025.1544151 (PMC12960119; doi:10.3389/fpubh.2025.1544151)
Supplement: Supplementary file 1 [file Data_Sheet_1.docx]

APPENDIX A: SAMPLE DEMOGRAPHICS

The average age of the sample is 43.20 years (s.d.=12.83). The sample is 1.7% Aboriginal/ Torres Strait Islander and 80.20% were born in Australia. Unsurprisingly, 99.4% of respondents identify as male, with the rest identifying as female, transgender, or “genderqueer.” Another 93.5% identify as heterosexual. In terms of relationship status, 16.9% are single; 7.5% are in a relationship, but not living together; 11.4% are living with a partner; 3.0% are engaged, 57.7% are married; 1.3% are divorced; 1.9% are separated; and 0.3% are widowed. At the time of data collection, some 86.90% were in paid employment. Crucially for purposes of the present study, 24.5% of sample respondents have had suicidal thoughts in the past 12 months. More detailed demographics can be found in AFIS (2021).

APPENDIX B: LIST OF STUDY VARIABLES

TABLE 1:

|  | Item(s) | Measurement |
| --- | --- | --- |
| Criterion Variable:  **Suicide** | Have you seriously considered attempting suicide in the past 12 months? | 0=No  1=Yes |
| Step 1  Predictor Variable:  **COVID-19 Job Loss** | Did any of the following happen to you as a result of the coronavirus restrictions? I lost my job, but my workplace did not cease operating. | 0=No  1=Yes |
| Step 2 Covariate:  **Job Satisfaction** | Taking everything into consideration, how do you feel about your job as a whole? | 1=Very dissatisfied  2=Moderately dissatisfied  3=Slightly dissatisfied  4=Neither dissatisfied nor satisfied  5=Slightly satisfied  6=Moderately satisfied  7=Very satisfied |
| Step 3 Additional Covariates:  **Aboriginal**  **Living Alone**  **Age** | Do you identify as Aboriginal, Torres Strait Islander or both?  I live alone.  What is your date of birth? | 0=No  1=Yes  0=No  1=Yes  Scale measurement |
| Step 4 Additional Covariates:  **Depression (past 12 months)**  **Anxiety (past 12 months)**  **Other Mental Health Conditions (past 12 months)**  **Risk-Taking Score**  **Subjective Wellbeing Score** | Have you been treated for depression in the past 12 months?  Have you been treated for anxiety in the past 12 months?  Have you been treated for any other mental health condition in the past 12 months?  How willing are you to take risks?  Composite of satisfaction with: standard of living, health, what you are achieving in life, personal relationships, how safe you feel, feeling part of your community, your future security | 0=No  1=Yes  0=No  1=Yes  0=No  1=Yes  0=Not at all willing to take risks  to  10=Very willing to take risks  Scale measurement |
| Step 5 Additional Covariates:  **Self-Rated Health**  **Minutes of Sleep (weekdays)**  **Minutes of Sleep (weekends)**  **BMI**  **Whether respondent has been diagnosed with a health condition (past 12 months)** | In general, would you say your health is:  In the last seven days, how many minutes did you usually sleep each night (weekdays)?  In the last seven days, how many minutes did you usually sleep each night (weekends)?  Derived from respondent height and weight  Respondent has been diagnosed with any health condition in the past 12 months? | 1=Poor  2=Fair  3=Good  4=Very good  5=Excellent  Scale measurement  Scale measurement  Scale measurement  0=No  1=Yes |
| Step 6 Additional Covariate:  **Alcohol Consumption Score** | Composite of alcohol frequency, number of standard drinks on drinking day, six or more drinks in one session, can’t stop drinking, failed expectations due to drinking, need drink in the morning, feel guild/regret, can’t remember after drinking, injury due to drinking, others concerned about drinking | Scale measurement |
| Step 7 Additional Covariates:  **Overall Life Optimism**  **Job Stability Optimism**  **Financial Situation Optimism**  **Housing Optimism**  **Relationships Optimism**  **Mental Health Optimism**  **Physical Health Optimism**  **Adaptation Optimism** | How optimistic or pessimistic do you feel about:  Your life overall?  Your employment and job stability?  Your financial situation?  Your housing?  Your relationships?  Your mental health?  Your physical health?  Your ability to adapt to future challenges? | 1=Very pessimistic  2=Somewhat pessimistic  3=Neither optimistic nor pessimistic  4=Somewhat optimistic  5=Very optimistic |
| Step 8 Additional Covariates:  **Language or Accent**  **Skin Color**  **Age**  **Disability**  **Religious Beliefs**  **Cultural Background**  **Mental Health Problems**  **Sexual Identity**  **Body Appearance**  **Sex**  **Gender** | In the last six months, you have been treated unfairly or badly because of your:  Language or accent?  Skin color?  Age?  Disability?  Religious beliefs?  Cultural background?  Mental health problems?  Sexual identity or same sex attraction?  Body size, shape, or physical appearance?  Sex (i.e., sexism)?  Gender identity (i.e., transgender, gender non-conforming)? | 0=No  1=Yes |

APPENDIX C: STATISTICAL TECHNIQUE

Given the binary nature of the dependent variable and the configuration of predictors, either multivariate probit or logistic regression could have been used as a statistical technique. Pampel (2000: 68) recommends the latter over the former because “probit analysis does not allow calculation of the equivalent of odds ratios, and makes calculation of changes in probabilities more difficult than in logistic regression.” Therefore, we employed multivariate binary logistic regression analysis to predict suicidal thoughts.

APPENDIX D: FULL BINARY MULTIVARIATE LOGISTIC REGRESSION RESULTS PREDICTING SUICIDAL THOUGHTS (N=963)

TABLE 2:

| **Predictor** | **B** | **Standard Error** | **Wald** | **P-value** | **Exp(B)** | **95%C.I. Lower** | **95%C.I. Upper** |
| --- | --- | --- | --- | --- | --- | --- | --- |
| COVID-19 Job Loss | 1.02 | .45 | 5.07 | .024 | 2.77 | 1.14 | 6.70 |
| Job Satisfaction | -.08 | .06 | 2.24 | .135 | .92 | .83 | 1.03 |
| Aboriginal | -1.30 | .84 | 2.37 | .124 | .27 | .05 | 1.43 |
| Living Alone | .29 | .28 | 1.04 | .308 | 1.34 | .77 | 2.33 |
| Age | -.01 | .01 | 1.47 | .225 | .99 | .97 | 1.01 |
| Depression Past 12 Months | .68 | .26 | 7.06 | .008 | 1.97 | 1.19 | 3.24 |
| Anxiety Past 12 Months | .33 | .28 | 1.41 | .236 | 1.39 | .81 | 2.39 |
| Other Mental Health Condition Past 12 Months | .31 | .36 | .72 | .396 | 1.36 | .67 | 2.76 |
| Risk Taking Score | .01 | .05 | .01 | .905 | 1.01 | .92 | 1.10 |
| Subjective Wellbeing Score | -.02 | .01 | 6.40 | .011 | .98 | .96 | 1.00 |
| Self-Rated Health | -.02 | .13 | .04 | .848 | .98 | .76 | 1.25 |
| Minutes Sleep (Weekday) | .00 | .00 | .95 | .330 | 1.00 | 1.00 | 1.01 |
| Minutes Sleep (Weekend) | -.00 | .00 | .70 | .404 | 1.00 | 1.00 | 1.00 |
| BMI | .01 | .02 | .53 | .467 | 1.01 | .98 | 1.05 |
| No Health Conditions Past 12 Months | .19 | .25 | .56 | .455 | 1.21 | .74 | 1.97 |
| Alcohol Consumption Score | -.00 | .01 | .00 | .956 | 1.00 | .97 | 1.03 |
| Overall Life Optimism | .03 | .14 | .05 | .829 | 1.03 | .79 | 1.35 |
| Job Stability Optimism | .03 | .12 | .05 | .827 | 1.03 | .81 | 1.31 |
| Financial Situation Optimism | .01 | .14 | .01 | .926 | 1.01 | .77 | 1.34 |
| Housing Optimism | .26 | .13 | 3.83 | .050 | 1.29 | 1.00 | 1.67 |
| Relationships Optimism | -.25 | .10 | 6.22 | .013 | .78 | .66 | .95 |
| Mental Health Optimism | -.61 | .13 | 22.49 | <.001 | .55 | .43 | .70 |
| Physical Health Optimism | .20 | .12 | 2.84 | .092 | 1.22 | .97 | 1.55 |
| Adaptation Optimism | -.09 | .12 | .55 | .459 | .91 | .72 | 1.16 |
| Treated Unfairly Language or Accent | 1.23 | .83 | 2.18 | .140 | 3.41 | .67 | 17.42 |
| Treated Unfairly Skin Color | -.80 | .69 | 1.34 | .247 | .45 | .12 | 1.73 |
| Treated Unfairly Age | .14 | .35 | .16 | .687 | 1.15 | .58 | 2.28 |
| Treated Unfairly Disability | -.78 | .87 | .82 | .365 | .46 | .08 | 2.49 |
| Treated Unfairly Religious Beliefs | -.54 | .75 | .50 | .478 | .59 | .13 | 2.57 |
| Treated Unfairly Cultural Background | -1.10 | .86 | 1.65 | .200 | .33 | .06 | 1.78 |
| Treated Unfairly Mental Health Problems | .80 | .42 | 3.66 | .056 | 2.23 | .98 | 5.06 |
| Treated Unfairly Sexual Identity | -.18 | .57 | .10 | .755 | .84 | .27 | 2.57 |
| Treated Unfairly Body Appearance | -.32 | .33 | .95 | .330 | .73 | .38 | 1.38 |
| Treated Unfairly Sex | .07 | .51 | .02 | .889 | 1.07 | .40 | 2.92 |
| Treated Unfairly Gender | 1.57 | .96 | 2.70 | .101 | 4.82 | .74 | 31.56 |
| Constant | 1.08 | 1.04 | 1.07 | .300 | 2.94 | N/A | N/A |

Dependent Variable: Suicidal Thoughts

-2 Log Likelihood=778.33

Cox & Snell R Squared=.19

Nagelkerke R Squared=.29

Hosmer & Lemeshow=3.94 (p=.86)
